# 

**Published:** 1894-02-03

**Authors:** 


**Notice to Advertisers and Subscribers.**

All Advertisements, Orders for Copies of The Hospital, and Business Communications should be addressed to The Publisher, *not to the Editor*, The Hospital, 428, Strand, W.C.

The Cost of Subscription, post free, is as follows:—Per week, 2½d.; per quarter, 2s. 8d.; per half-year, 5s. 3d.; per annum, 10s. 6d. Foreign:—Per Annum, 12s. 8d.; payable, in all cases, in advance.

**OUR NEW OFFICES.**

428, Strand, W.C., will henceforth be the Office of this Journal.

**Notices of Births, Deaths, and Marriages** are inserted in this column at a charge of 2s. 6d. for an announcement not exceeding four lines.

**NOTICE TO CORRESPONDENTS.**

All MS., Letters, Books for Review, and other matters intended for the Editor should be addressed The Editor, The Lodge, Porchester Square, London, W.

The Editor cannot undertake to return rejected MS., even when accompanied by stamped directed envelope.

**"Burdett's Hospital Annual," 1893.**

Fourth year of publication. 700 pp. Boards, gilt lettering. A most complete guide to British, Colonial, Indian, and American Hospitals, Dispensaries, Nursing and Convalescent Institutions, and Asylums. Price 5s. Published by The Scientific Press (Limited), 428, Strand, W.C.

**NOTICE.—The Index for Vol. XIV. (ending Sept. 30th, 1893) is on sale at the Office of this Journal, price 2½d., post free.**

### Scraps and Gleanings.

It has been proposed to establish a new hospital for Lower Renfrewshire; further accommodation for patients being required

Mr. J. Whitaker Hunt, President of the Royal College of Surgeons, has been appointed to deliver the Hunterian oration next year.

The 33rd annual meeting of the working men's committee of the Hull Royal Infirmary took place recently. The record of subscriptions show some falling off.

Pending the question of a fever hospital for Eas'er Ross (Ross-shire) being remitted to the Public Health Committee, it is suggested to provide temporary and moveable accommodation for infectious cases in that district.

An exceedingly well-arranged and brilliant concert was held at the Dublin Royal Hospital for Incurables recently. The entertainment was provided for the delectation of the patients, and inaugurated by a lady life governor.

Everyone interested in the prosperity of the Edinburgh Royal Infirmary must wish success to the motion of which the Lord Provost has given notice, that henceforward the meetings of the managers should be open to representatives of the press.

During the past year 2,559 in-patients have been received at the Devonshire Hospital and Buxton Bath Charity. The financial position of the hospital has been, on the whole, satisfactorily maintained, the total receipts being greater than the total expenditure by £113.

In presenting the eleventh annual report of the Kendal Provident Dispensary, the committee stated that the work of the institution continues to be satisfactory. There is, however, some very slight falling off of subscriptions towards the funds of this dispensary.

The annual meeting of subscribers to the Ayr County Hospital has just taken place. The total income during the period under review was stated to be £2,717, and the outlay £2,809, leaving a deficit of £92. The work of the hospital was reported to continue in all its wonted efficiency.

During the year the whole drainage of this Institution has been overhauled at considerable cost, and increased lavatory accommodation has also been provided. The subscription list of the hospital has fallen off somewhat from the preceding twelve months, and, taking into account the expenses incurred above, the Board of Management are urgent for increased support in that direction.

The assent of the Local Government Board at Rhyl to the loan of £2,000 for the Infectious Diseases Hospital has been received, and at a special meeting of the Improvement Commissioners the necessary resolutions were adopted with the view of proceeding at once with the erection of the building.

At the annual general meeting of the Brighton Dispensary, which took place recently, it was stated that the number of patients (including those attended at their own homes) amounted to 14,235 during the year under review. The Western Branch endowment fund has benefited from various sources somewhat considerably.

The annual meeting of the Governors of the City Dispensary, the oldest medical charity in the City, was recently held. The report of the committee for the last year showed that 11,627 patients had been treated during the year, the ordinary income for which period had amounted to £1,318, and the expenditure to £1,251.

Last month a meeting took place of those interested in opposing the contemplated establishment at Perivale of a joint isolation hospital, for the accommodation of the parishes of Acton, Chiswick, and Hanwell, with the object of considering the steps to be taken to prevent the establishment of such an institution in the locality.

The fifth annual general meeting of the subscribers to the Budleigh Salterton Cottage Hospital, Exeter, took place recently, the report of which institution stated that the total cos of maintenance of establishment, patients, and staff for 1893 amounted to £190; a balance to the good of £42 remains on the balance-sheet.

The annual meeting of the governors of the Canterbury Medical Charity was held recently. The Committee had again the satisfaction of reporting on the continued success of this institution, During the year 1,559 patients had been benefitted. The financial statement showed receipts amounting to £461, and a balance in hand of £30.

From the last report of the Committee of the Seaford Convalescent Home, to which we have previously made some slight allusion, one gathers that the great wave of general depression which has passed over the country has affected the finances of the home, and, further, it is stated that unless generous support is forthcoming, the great benefits conferred by this institution must be in a serious degree lessened.

In anticipation of Hospital Sunday, the Aberdeen Infirmary board of directors have drawn attention to the financial position of the hospital at the close of 1893. They anticipate a deficit of £1,800. They claim that the Aberdeen Infirmary may be classed amongst the most economically conducted hospitals in the kingdom. At the same time the expenditure must rather increase than diminish, owing to the large extensions.

The first annual meeting of the subscribers and friends of the Manchester Cancer Pavilion and Home has just taken place. During the first six months of its existence six beds had been opened at the institution, during the remainder of the year twenty. There had been fifty-seven patients in the course of the period under review. On the year's working there was a balance of £500 against their accounts, owing to the initial expenses of such an institution being necessarily very heavy.

### Medical Vacancies and Appointments.

### APPOINTMENTS.

W. Barber, appointed Medical Officer for the Kilburn District of the parish of St. John, Hampstead. J. H. Cropper, L.R.C.P., L.R.C.S., appointed Clinical Assistant to the Royal Westminster Ophthalmic Hospital. C. J. Fowler, L.D.S.R.C.S.Eng., appointed Consulting Dental Surgeon to the Birmingham Children's Hospital. E. A. Gibson, M.B., C.M.Glasg., appointed House-Surgeon to the Paddington Green Children's Hospital. W. H. Gravely, L.R.C.P.Lond., M.R.C.S.Eng., appointed Temporary Medical Officer for the Cowfold District of the Cuckfield Union. J. Leach, M.A., M.B., C.M.Aberd., appointed House-Surgeon to the Manchester Southern Hospital for Women and Children; also House-surgeon to the Manchester Maternity Hospital. J. H. Lilley, M.A., M.D., M.R.C.S., appointed Honorary Medical Officer of the Provident Branch of the Hereford Dispensary. T. Lyle, M.D.Glasg., appointed Surgeon to the Newcastle Throat and Ear Hospital. J. G. Modlin, M.B., B.S.Dunelm, L.S.A.Lond., appointed Honorary Surgeon to the Monkwearmouth and Southwick Hospital, Sunderland. H. Montgomerie, M.D.Edin., reappointed Physician to the West Cornwall Dispensary and Infirmary. J. B. Montgomery, M.D.Glasg., F.R.C.P.Lond., reappointed Consulting Physician to the West Cornwall Dispensary and Infirmary. H. C. Moore, M.R.C.S.Eng., L.S.A., appointed Honorary Medical Officer to the Hereford Infirmary. H. C. Roberts, M.R.C.S.Eng., L.R.C.P.Lond., appointed Deputy Surgeon to the Great Northern Railway, Melton Mowbray. W. M. Robertshaw, M.B., C.M.Univ.Edin., appointed Medical Officer of Health to the Stocksbridge Local Board, Yorkshire. J. Symons M.R.C.S.Eng., reappointed Surgeon to the West Cornwall Dispensary and Infirmary. H. H. Tipping, M.R.C.S., L.R.C.P.Lond., appointed Resident Medical Officer to the Birmingham Workhouse Infirmary. J. H. Traquair, M.B., C.M.Edin., appointed Junior Assistant Medical Officer to the Staffordshire County Asylum, Burntwood, Lichfield. J. B. Welch, M.B.Lond., M.R.C.S.Eng., appointed Honorary Consulting Physician to the Birmingham Children's Hospital. W. A. Wills, M.D.Lond., M.R.C.P., appointed Physician to the North-Eastern Hospital for Children, Hackney.

### VACANCIES.

### Resident Medical Officers.

Carrickmacross Fever Hospital. Medical Officer. Salary £50 per annum. Election on February 6th, when candidates must personally attend.

Central London Ophthalmic Hospital, Gray's Inn Road, W.C. House Surgeon. Applications, with testimonials, to the Secretary, by February 5th.

Greenwich Union Infirmary. Second Assistant Medical Officer; unmarried. Salary £80 per annum, with board, lodging, and attendance. Applications to Samuel Saw, Clerk to the Guardians, Union Offices, Greenwich, by February 5th.

Ramsgate and St. Lawrence Royal Dispensary and Seamen's Infirmary. Medical Officer; unmarried; doubly qualified. Salary £120 per annum—£10 allowed for substitute for annual holiday—with furnished apartments, gas, firing, and attendance. Applications to the Secretary, by February 6th.

West Derby Union. Assistant Medical Officer for the Workhouse; doubly qualified. Salary £100 per annum, with first-class rations and apartments. Applications to Harris P. Cleaver, Union Clerk, Brougham Terrace, Liverpool, by February 6th.

### Non-Resident Medical Officers.

Axminster Union. Medical Officer for four districts. Salary £72 16s. 8d. per annum; midwifery and surgical operations extra. Applications to W. Forward, Clerk, by February 7th.

Brighton Throat and Ear Hospital, 23, Queen's Road, Brighton. House Surgeon. Salary £52 per annum. Applications to the Secretary, by February 5th.

Dental Hospital of London, Leicester Square, W.C. Assistant Dental Surgeon. Applications to J. Francis Pink, Secretary, by March 12th.

Kilburn, Maida Vale, and St. John's Wood Dispensary.—Vacancy on the Honorary Medical Staff. Applications to the Secretary, 13, Kilburn Park road, by February 14th.

New Hospital for Women, 144, Euston Road, N.W.—Lady Dispenser. Salary, £90 per annum. Applications to the Secretary, by February 17th.

Owens College, Manchester. Professor of Zoology. Applications to the Council of the College, under cover to the Registrar, by April 3rd.

Royal Pimlico Dispensary, Buckingham Palace Road, S.W. Attending Medical Officer; must reside in the district. Applications and testimonials to the Secretary, by February 5th.

Westminster Hospital Medical School. Lecturer on Bacteriology. Applications to the Dean, Mr. Spencer, by February 6th.

